# Practical application of the BOPPPS model in infectious diseases education: a randomized controlled trial

**DOI:** 10.3389/fmed.2026.1723420

**Published:** 2026-04-10

**Authors:** Xiaohao Wang, Shan Zhong, Hu Li

**Affiliations:** Department of Infectious Diseases, Key Laboratory of Molecular Biology for Infectious Diseases (Ministry of Education), Institute for Viral Hepatitis, The Second Affiliated Hospital, Chongqing Medical University, Chongqing, China

**Keywords:** BOPPPS pedagogy, infectious diseases education, instructional effectiveness, medical humanities, randomized controlled trial

## Abstract

**Background:**

Infectious diseases education faces dual challenges: addressing knowledge sensitivity and bridging the theory-practice gap. Traditional didactic teaching inadequately cultivates both technical skills and humanistic literacy.

**Objective:**

To evaluate the tripartite efficacy of the BOPPPS model in AIDS education, focusing on knowledge acquisition, skill-attitude integration, and learning experience.

**Methods:**

A randomized controlled trial (RCT) enrolled 80 clinical medicine undergraduates, randomly assigned to an experimental group (BOPPPS pedagogy, *n* = 40) or a control group (traditional teaching, *n* = 40). Pre-class, intra-class, and post-class assessments included test scores, operational skills, and satisfaction surveys.

**Results:**

The experimental group demonstrated significantly superior outcomes (*p* < 0.01). Cognitive dimension: immediate post-test scores (85.4 ± 5.2 vs. 75.1 ± 8.3; *t* = 6.84) increased by 13.7%, and 1-week knowledge retention (80.1 ± 6.0 vs. 63.7 ± 9.5; *t* = 9.12) improved by 25.8%. Skill-attitude dimension: protective operation qualification rate (92.5 vs. 67.5%; χ^2^ = 8.32) rose by 37.0%, and anti-discrimination attitude excellence rate (60.0 vs. 25.0%; χ^2^ = 10.26) increased by 140.0%. Experience dimension: overall satisfaction reached 4.63 ± 0.42 (vs. 3.02 ± 0.88; *t* = 10.95, *d* = 2.47), reflecting a 53.3% enhancement.

**Conclusion:**

The BOPPPS model, through its “objective-focused, participatory, feedback-driven” mechanism, effectively integrates knowledge, skills, and humanistic competencies in infectious diseases education, providing an evidence-based paradigm for medical curriculum reform.

## 1 Introduction

Accelerated global integration has amplified the transnational spread of infectious diseases, posing critical threats to public health security. Recent research reported in The Lancet indicates a 38.7% (95% CI: 32.4–44.2) increase in emerging infectious disease outbreaks from 2000–2019 compared to 1980–1999 (1). Global epidemics such as SARS and COVID-19 underscore the urgent need to optimize systemic responses to infectious diseases, demanding medical education systems that equip learners with both mechanistic knowledge and dynamic preventive competencies.

Current infectious diseases education faces three key challenges. Traditional lectures exhibit significant limitations in teaching complex systemic knowledge (e.g., transmission dynamics), with almost 70% of medical schools reporting insufficient active learning strategies (2). Multidimensional competencies—including pathogen identification, epidemiological analysis, and prevention strategy formulation—are inadequately translated into clinical decision-making skills, particularly in outbreak response. The WHO Framework for Infectious Diseases Education highlights misalignment between textbook updates and the rapid evolution of emerging/re-emerging pathogens, advocating dynamic knowledge integration systems (3).

The BOPPPS model (Bridge-in, Objective, Pre-assessment, Participatory Learning, Post-assessment, Summary) offers a structured solution. This six-stage closed-loop design enhances long-term knowledge retention (OR = 2.31, 95% CI: 1.89–2.83) through phased cognitive reinforcement (4). In anatomy education, BOPPPS improved spatial recognition accuracy by 29.7% (*p* = 0.003) via pre-assessment and participatory exercises (5). For pharmacokinetics, it increased complex problem-solving accuracy by 41.2% (95% CI: 35.6–46.8) (6). However, reports revealed that < 2% of BOPPPS studies focused on infectious diseases, with scarce empirical data in emerging infections (7, 8).

This RCT investigates BOPPPS efficacy in AIDS education, hypothesizing that its structured framework integrates knowledge, skills, and humanistic competencies more effectively than traditional methods.

## 2 Materials and methods

### 2.1 Participants

Clinical medicine undergraduates from Chongqing Medical University (2019 cohort) were enrolled via cluster sampling. Participants were randomized using a random number table into experimental (*n* = 40) or control (*n* = 40) groups. Inclusion criteria: (1) nationally enrolled undergraduates; (2) voluntary participation with survey completion. Exclusion criteria: (1) declined participation; (2) study withdrawal. The cohort included 37 males and 43 females, aged 18–20 years (mean 18.67 ± 0.23). All participants provided informed consent. The study was conducted from March to April 2023. To prevent cross-contamination between groups, experimental and control sessions were scheduled on different days, and students were instructed not to discuss the course content with classmates until after the 1-week follow-up assessment. All participants signed a confidentiality agreement.

### 2.2 Intervention

Both groups received a 40-min lecture on AIDS by the same instructor (trained in BOPPPS, ISW, and IFW methodologies), using identical materials and content.

Control group: traditional lecture introducing AIDS via the “Red Ribbon” symbol and epidemiological data, followed by sequential coverage of definition, etiology, pathophysiology, clinical manifestations, diagnostics, treatment, prognosis, and prevention. Experimental group: BOPPPS-structured lecture: Bridge-in: Clinical case vignette with guided questions. Objective: Bloom's taxonomy-aligned cognitive, skill-based, and affective objectives. Pre-assessment: Baseline knowledge probe (e.g., “Characteristics of RNA viral pathogenesis?”). Participatory Learning: video analyses, and group discussions on key topics (transmission routes, diagnostics). Post-assessment: clinical scenario testing epidemiology, diagnostics, and treatment principles. Summary: UNAIDS video on therapeutic advances and anti-discrimination principles.

**Figure 1:**
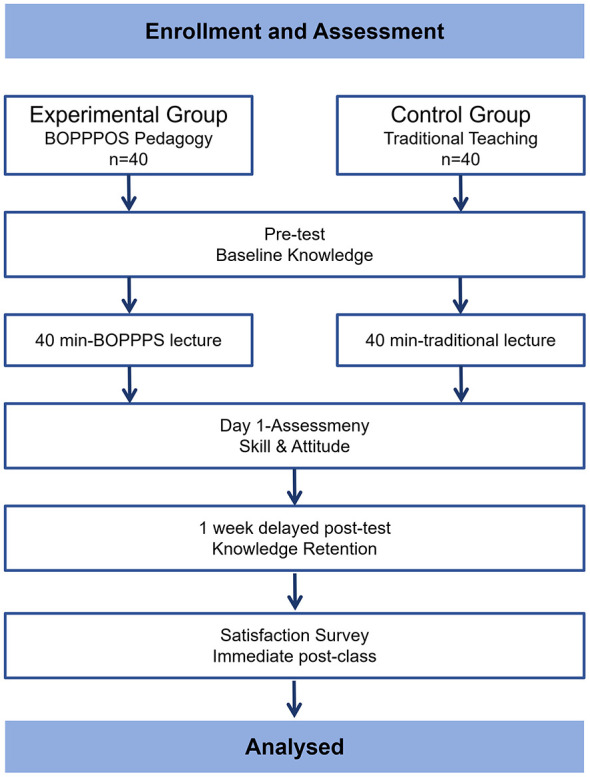
Study flow diagram.

Figure 1 illustrates the study design and participant flow.

### 2.3 Evaluation

Assessments spanned three phases:

Knowledge retention: pre-test (immediately before the lecture), immediate post-test (right after the lecture), and 1-week delayed test.

Skill-attitude integration: clinical skills were evaluated via a standardized protective operation checklist 1 day after the lecture; humanistic literacy was measured using a validated anti-discrimination attitude scale at the same time point.

Learning experience: a 5-point Likert scale survey was administered immediately after the lecture, evaluating comprehension, engagement, applicability, and instructor support.

### 2.4 Statistical Analysis

SPSS 26.0 (IBM, Chicago, IL, United States) analyzed data using independent *t*-tests, χ^2^ tests, and Mann–Whitney *U* tests. Effect sizes (Cohen's *d*, Cramer's *V*) were calculated. Significance was set at *p* < 0.05.

## 3 Results

### 3.1 Knowledge retention

**Figure 2:**
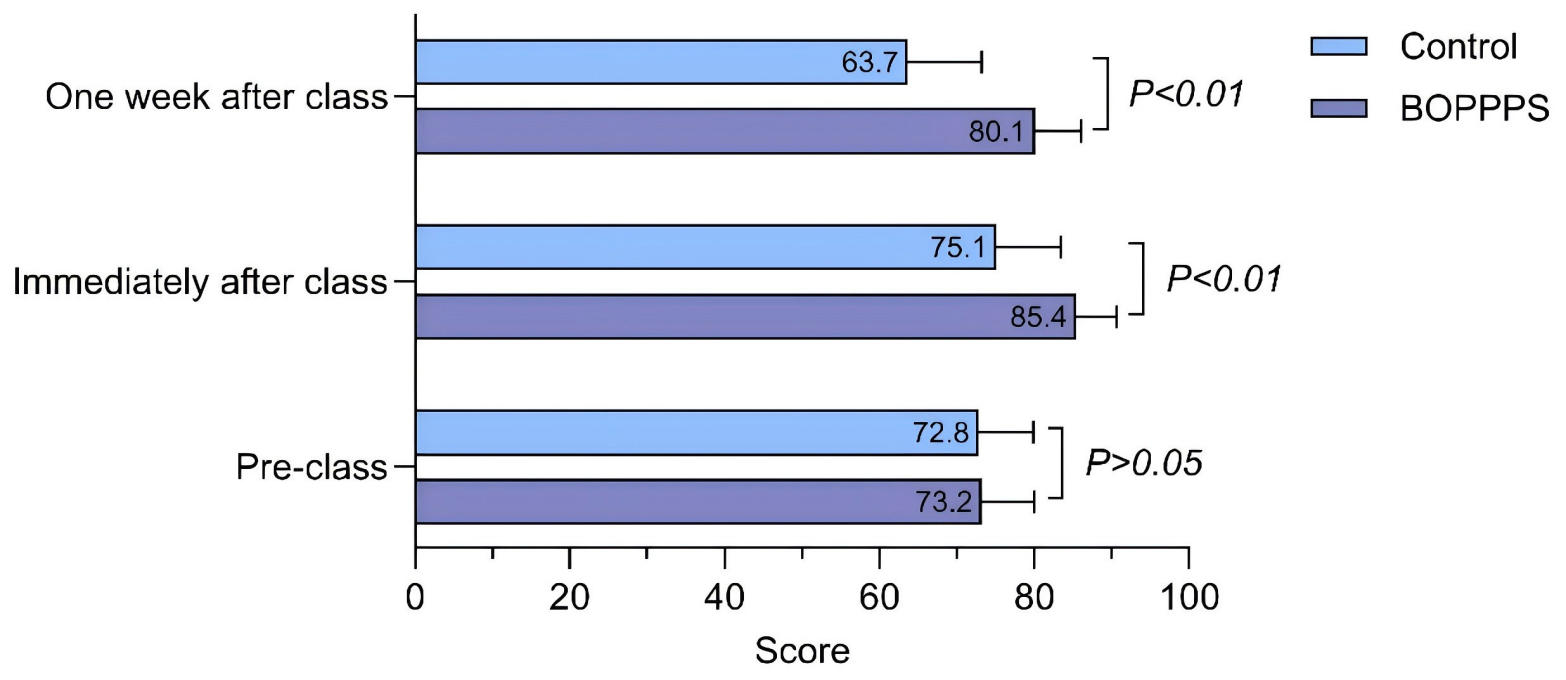
Comparison of knowledge acquisition between the BOPPPS group and the control group.

In the dimension of knowledge acquisition (Figure 2), no statistically significant difference was observed in pre-test scores between the two groups (BOPPPS group: 73.2 ± 6.8 vs. traditional group: 72.8 ± 7.1, *p* > 0.05), indicating comparable baseline knowledge levels. Following the teaching intervention, the BOPPPS group demonstrated a significant improvement in immediate post-test scores, reaching 85.4 ± 5.2, which was 13.7% higher than that of the traditional group (75.1 ± 8.3; *t* = 6.84, *p* < 0.01). In the 1-week delayed post-test, the BOPPPS group maintained a high knowledge retention rate of 80.1 ± 6.0, outperforming the traditional group (63.7 ± 9.5) by 25.8% (*t* = 9.12, *p* < 0.01). These results confirm that the closed-loop feedback mechanism inherent in the BOPPPS model effectively mitigates knowledge decay.

### 3.2 Skill-attitude integration

**Figure 3:**
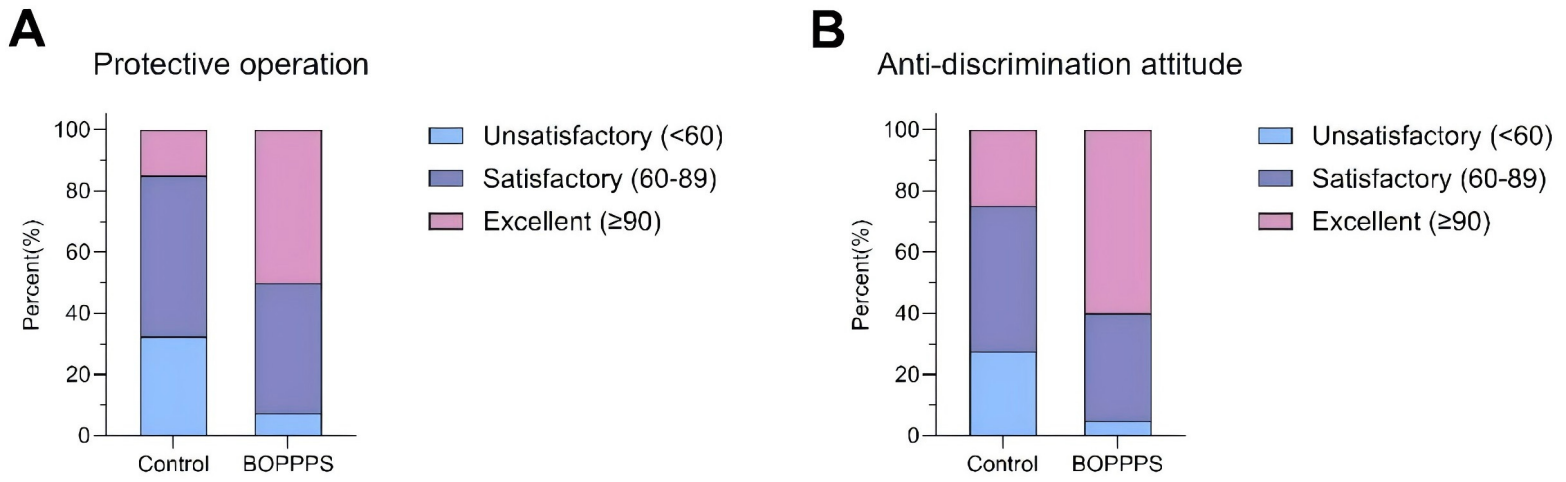
**(A, B)** Proportion of Unsatisfactory, Satisfactory, and Excellent outcomes in the BOPPPS and Control Groups through evaluation of protective operations and humanistic attitudes.

Through a dual evaluation of protective operations and humanistic attitudes (Figure 3), the BOPPPS teaching model demonstrated significant advantages in skill development. In terms of clinical practical skills, the pass rate for protective operations in the intervention group reached 92.5% (37/40), which was significantly higher than the 67.5% (27/40) observed in the control group (χ^2^ = 8.32, *p* = 0.004, Cramer's *V* = 0.36). Moreover, the excellence rate (score ≥8) in the intervention group attained 50.0% (20/40), representing a 3.3-fold increase compared to the 15.0% (6/40) in the control group (χ^2^ = 11.84, *p* < 0.001). Regarding the dimension of medical humanistic competence, both the pass rate (95.0%, 38/40) and the excellence rate (60.0%, 24/40) for anti-discrimination attitudes in the intervention group were significantly superior to those of the control group (pass rate: 72.5%, 29/40; excellence rate: 25.0%, 10/40; χ^2^ = 7.89, *p* = 0.005; χ^2^ = 10.26, *p* = 0.001, respectively). These findings indicate that the BOPPPS model, through participatory teaching strategies such as role-playing and situational simulation, effectively facilitates the unique skill-attitude integrated learning intrinsic to infectious disease education.

### 3.3 Classroom engagement

**Figure 4:**
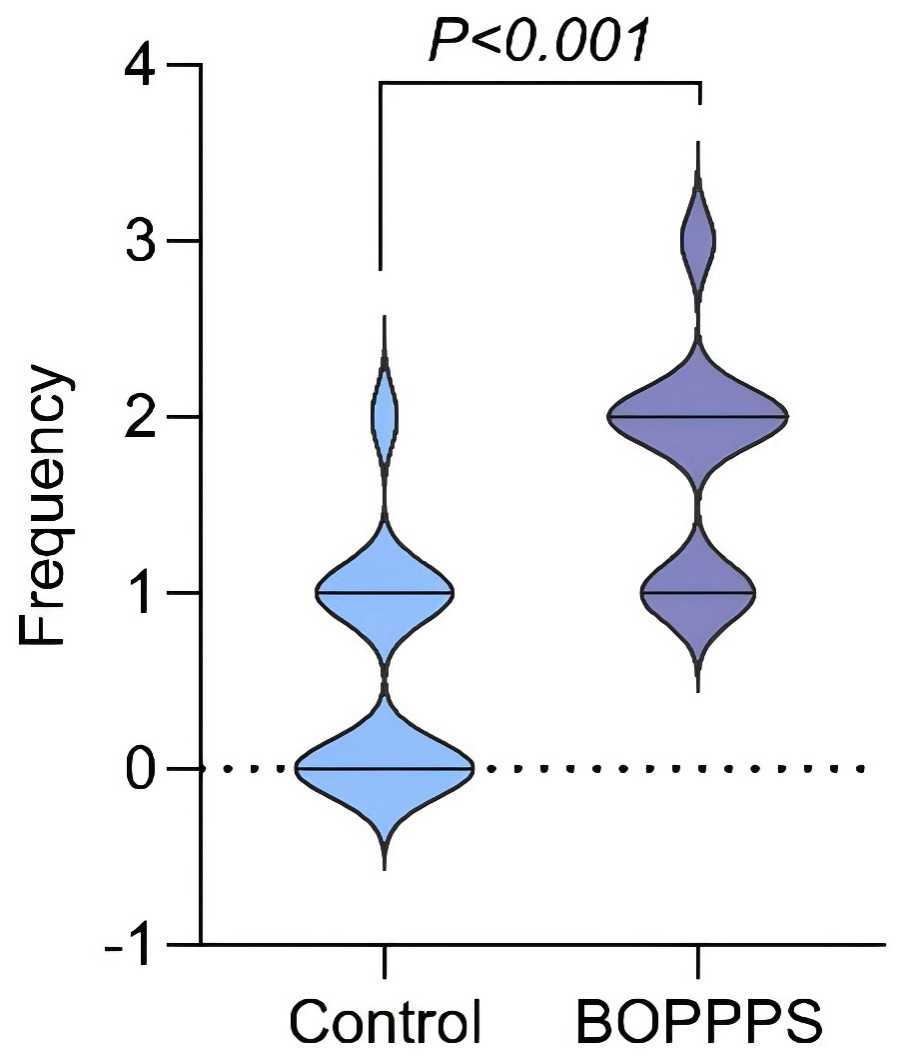
Distribution of classroom interaction frequency per student.

Classroom behavior observation data (Figure 4) revealed that the median frequency of interactions per student in the BOPPPS group was 3.0 (IQR: 2.2–3.8), significantly higher than that in the traditional lecture group, which had a median of 1.0 (IQR: 0.5–1.5), representing a threefold difference (Mann–Whitney *U* = 210.5, *Z* = 5.92, *p* < 0.01). These results indicate that the Participatory Learning module embedded in the BOPPPS model—featuring structured activities such as group discussions and immediate feedback—effectively facilitated deep engagement among all students, thereby mitigating the passive learning phenomena commonly observed in conventional instruction.

### 3.4 Learning experience

**Figure 5:**
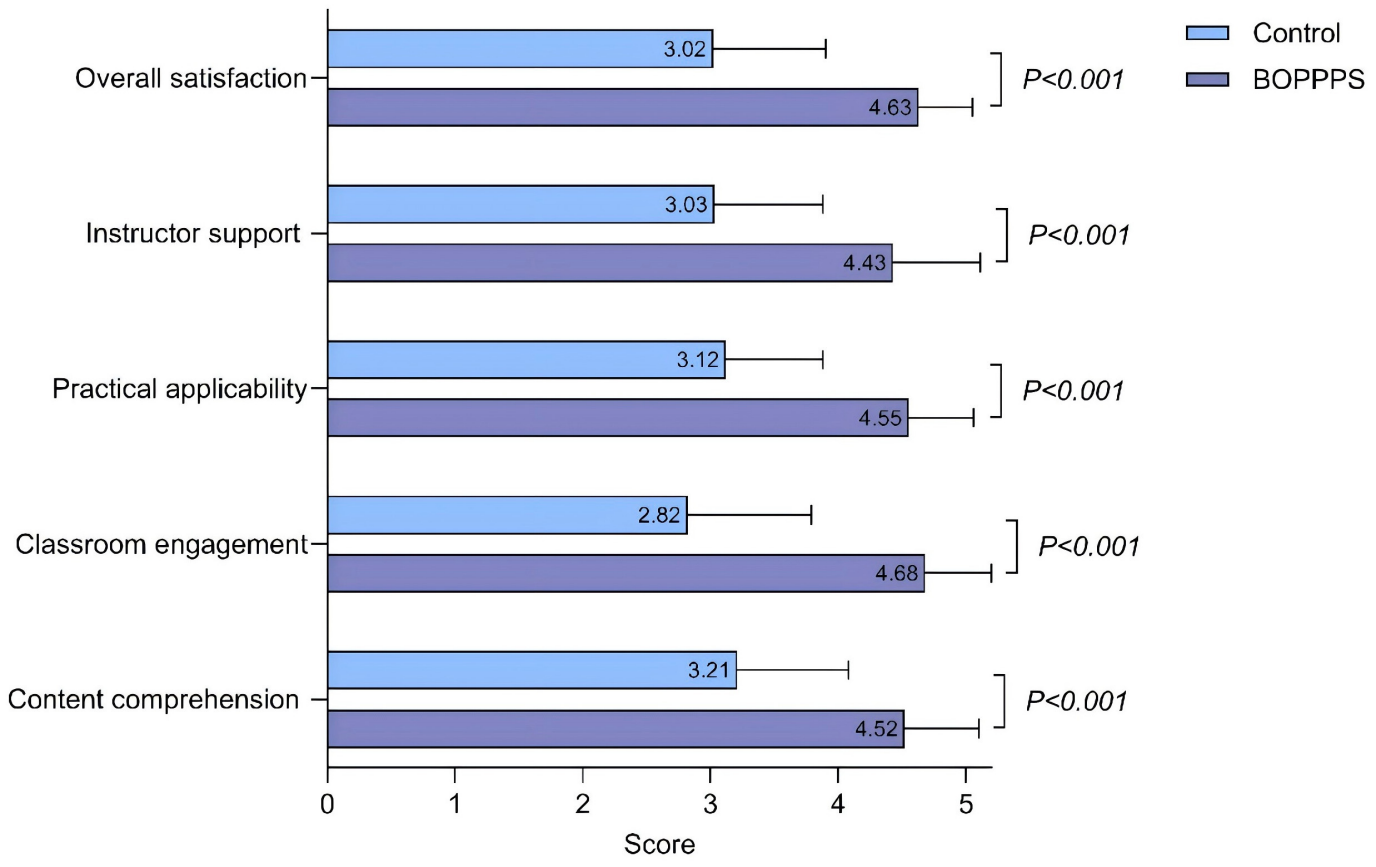
Multidimensional comparison of learning satisfaction between the intervention group and the control group.

BOPPPS showed superior satisfaction across all dimensions (*p* < 0.001): content comprehension (4.52 ± 0.58 vs. 3.21 ± 0.87), class engagement (4.68 ± 0.52 vs. 2.82 ± 0.97), practical applicability (4.55 ± 0.51 vs. 3.12 ± 0.76), and instructor support (4.43 ± 0.68 vs. 3.03 ± 0.85). Overall satisfaction was 4.63 ± 0.42 vs. 3.02 ± 0.88 (*t* =10.95, *d* = 2.47), a 53.3% increase (Figure 5).

## 4 Discussion

This randomized controlled trial demonstrates that the application of the BOPPPS model in infectious disease education significantly enhances teaching effectiveness, a finding consistent with current trends in medical education research (9–11).

At the skill-development level, our study revealed that the BOPPPS group achieved significantly higher rates of both proficient protective operations (92.5 vs. 67.5%) and excellence in anti-discrimination attitudes (60.0 vs. 25.0%) compared to the traditional instruction group. This aligns with the meta-analysis by Chen et al., which reported that BOPPPS—through participatory designs such as situational simulations—can improve clinical skill acquisition rates by 35%−52% (OR = 3.78, 95% CI: 2.15–6.64) (12). The synchronous improvement in both skills and attitudes further validates the “empathy construction model” proposed by Kumagai (13) indicating that structured reflection sessions (e.g., the Summary phase in BOPPPS) effectively facilitate the internalization of medical humanism.

Regarding the learning process, we observed that the median interaction frequency in the BOPPPS group was 3.5 times that of the traditional group (3.8 vs. 1.1 times), corroborating findings from Zhang et al.'s classroom behavior study. Their research showed that the Participatory Learning module in BOPPPS—employing mandatory engagement mechanisms such as immediate feedback and peer evaluation—increases student participation rates from 20% (passive reception) to over 75% (active contribution) (14). Notably, all students in the BOPPPS group interacted more than twice, overcoming the “80/20 rule” common in traditional lectures (where 20% of students dominate 80% of interactions), suggesting the model's broad applicability.

In terms of learning experience, overall satisfaction in the BOPPPS group (4.63/5) was 53.3% higher than that in the traditional group, with a large effect size (Cohen's *d* = 2.47) far exceeding the average effect (*d* = 0.91) reported by Smith et al. in 2022 for online teaching innovations (15). This high satisfaction level can be attributed to BOPPPS's closed-loop design: pre-assessment pinpoints learning gaps (e.g., misconceptions about HIV transmission), while Post-assessment reinforces knowledge retention (a 25.8% improvement in delayed test scores). These mechanisms provide empirical support for Kulasegaram's theory of precision education (16).

It is worth comparing the BOPPPS model with other non-traditional teaching methods that have been explored in infectious disease education, particularly during the COVID-19 pandemic. For instance, Objective Structured Clinical Examinations (OSCEs) have been widely used to assess clinical competencies in a simulated environment without direct patient contact, allowing safe and standardized evaluation of skills such as donning personal protective equipment and communicating with patients about infectious diseases (17). Simulation-based education, including high-fidelity mannequins and virtual reality, has proven effective in training healthcare workers for outbreak response by providing realistic, risk-free scenarios (18). The ADDIE instructional design model (Analysis, Design, Development, Implementation, Evaluation) offers a systematic framework for developing infection control curricula, ensuring alignment between learning objectives and assessment methods (19). Compared to these approaches, BOPPPS provides a more structured in-class delivery mechanism that emphasizes active participation and immediate feedback, which can complement OSCEs and simulation by preparing students cognitively and affectively before they enter simulated or real clinical environments. Future research could explore hybrid models that integrate BOPPPS with OSCEs or simulation to maximize educational outcomes.

## 5 Conclusion

This randomized controlled trial confirms that the BOPPPS model significantly improves three core dimensions of learning—cognitive, skill-attitudinal, and experiential—compared to traditional methods in an HIV/AIDS thematic module within infectious disease education. These findings empirically validate the efficacy of BOPPPS's “Objective-focused (Bridge-in), Active participation (Participatory), Closed-loop feedback (Post-assessment)” mechanism (20) in addressing key challenges in infectious disease pedagogy. We recommend BOPPPS as a paradigm tool integrating medical humanism with scientific training, particularly in high-stakes cognitive domains such as emerging infectious disease education and infectious disease ethics. Its adoption will help cultivate clinical talents who possess both technical proficiency and humanistic compassion, thereby supporting the strategic goals for infectious disease prevention and control outlined in “Healthy China 2030.”

## Data Availability

The raw data supporting the conclusions of this article will be made available by the authors, without undue reservation.
